# Editorial Comment to Local advanced adrenocortical cancer with a long‐term recurrence‐free survival treated with complete surgical excision and adjuvant therapy with a very low‐dose mitotane

**DOI:** 10.1002/iju5.12215

**Published:** 2020-08-29

**Authors:** Koshiro Nishimoto

**Affiliations:** ^1^ Department of Uro‐Oncology Saitama Medical University International Medical Center Hidaka Saitama Japan

Tokura *et al*. reported a case with adrenocortical carcinoma (ACC) which invaded to the liver and was treated with right adrenalectomy, right hemi‐hepatectomy following adjuvant therapy using low‐dose mitotane.^1^ As results, the case survived for 90 months. In Japan, like the United States and European countries, mitotane is the only agent that approved for the treatment of high stage ACC. Although, the pharmacological mechanism of mitotane leading the adrenolytic effect is not fully elucidated, mitotane and its metabolites seem to inhibit mitochondrial function and production of steroidogenic enzymes in adrenocortical cells.^2^, ^3^, ^4^ It is recommended that the dose of mitotane is controlled to a blood level of 14–20 mg/L despite the regular measurements are not approved in Japan. Low‐dose treatment with mitotane, which do not require measurements of blood level, might be a good option as an adjuvant treatment for adrenocortical carcinoma as shown in previous reports.^5^ Further accumulation of case reports is desirable for the exceedingly rare type of carcinoma: ACC.

## Conflict of interest

The author declares no conflict of interest.
